# Stress cardiomyopathy associated with area postrema syndrome as a presentation of neuromyelitis optica: case report

**DOI:** 10.1186/s12883-020-01784-3

**Published:** 2020-05-21

**Authors:** Sungsik An, Hyeo-il Ma, Jooyeon Song, Hong-Mi Choi, Young Eun Kim

**Affiliations:** 1 Department of Neurology, Hallym University Sacred Heart Hospital, Hallym University College of Medicine, Anyang, South Korea; 2 Hallym Neurological Institute, Hallym University College of Medicine, Anyang, South Korea; 3 Department of Cardiology, Hallym University Sacred Heart Hospital, Hallym University College of Medicine, Anyang, South Korea

**Keywords:** Neuromyelitis optica, Stress cardiomyopathy, Takotsubo cardiomyopathy, Area postrema syndrome, Aquaporin-4 antibody

## Abstract

**Background:**

Stress cardiomyopathy (Takotsubo cardiomyopathy) is very rare in the central nervous system (CNS) demyelinating disorders. Although this dysfunction of the heart-brain axis has been reported in several case series related to multiple sclerosis (MS), stress cardiomyopathy by neuromyelitis optica (NMO), which is rarer CNS demyelinating disorder than MS, is extremely rare. Herein, we report a case of stress cardiomyopathy associated with a medullary lesion as a presentation of NMO.

**Case presentation:**

A 30-year-old woman was treated by veno-arterial extracorporeal membrane oxygenation due to catastrophic cardiopulmonary dysfunction after prolonged and unexplained nausea, vomiting, and cough. Myoclonus on the limbs developed afterward. Taken with suspicion of area postrema syndrome (APS), the brain MRI showed a demyelinating lesion in the medulla oblongata. APS and severe heart failure by stress cardiomyopathy were completely resolved by ECMO and hydrocortisone therapy. However, the CNS demyelinating lesion recurred after 1 month. The patient was diagnosed with NMO evident by the presence of aquaporin-4 antibody; Steroid therapy improved her symptoms.

**Conclusion:**

NMO should be considered as one of the differential diagnoses in patients with APS preceding severe cardiopulmonary distress.

## Main text

### Background

A rare but relevant association between stress cardiomyopathy (Takotsubo cardiomyopathy) and multiple sclerosis (MS) has been reported in several case series [1–4]. Neuromyelitis Optica (NMO) is a rarer autoimmune demyelinating disorder of the central nervous system (CNS) by a different immunological mechanism with MS. Herein, we on report a patient with NMO who presented with area postrema syndrome (APS) and a catastrophic cardiopulmonary crisis by stress cardiomyopathy.

### Case presentation

**Fig. 1:**
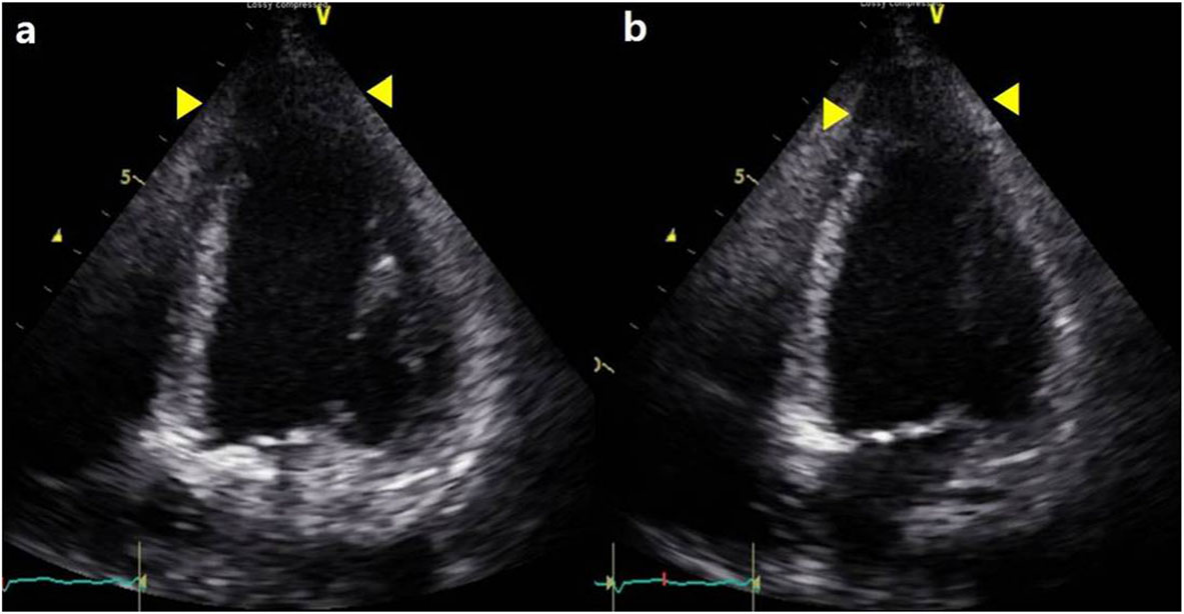
**a**, **b** Thoracic echocardiography showing akinesia on the base to the mid left ventricle (left, end-diastole; right, end-systole)

A 30-year-old woman without underlying disease suffered from unexplained nausea, vomiting, and cough without fever. After 1 month, she was admitted to the emergency department with suddenly deteriorated dyspnea and myoclonic jerks on all limbs. At admission, the chest X-ray showed multifocal patchy ground-glass opacity in the bilateral lungs. ST elevation of the inferior leads on the electrocardiography and increased cardiac markers (creatine kinase-MB, 33.1 ng/mL; troponin I, 10989.0 pg/mL, and N-terminal-pro b-type natriuretic peptide, 3837 pg/mL) were observed. The echocardiography showed severe left ventricular (LV) systolic dysfunction (LV Ejection fraction, LVEF 13%) and regional wall motion abnormalities (Fig. 1a,b). Coronary angiography conducted to exclude ischemic etiology was normal. Veno-arterial extracorporeal membrane oxygenation (ECMO) was performed to maintain her vitality under the suspicion of stress cardiomyopathy or acute myocarditis. After 2 days of ECMO with the administration of a vasopressor, hydrocortisone, and antibiotics, dyspnea and lung infiltration were rapidly improved. After 12 days, the LVEF was improved to 40% and was normal after 1 month. Cardiac magnetic resonance imaging (MRI) showed a high signal intensity in the T2-weighted image suggesting focal edema at 1 month after ECMO therapy. Pheochromocytoma was not detected on the abdominal computed tomography (CT) scan and in the 24-h urine collection.

**Fig. 2:**
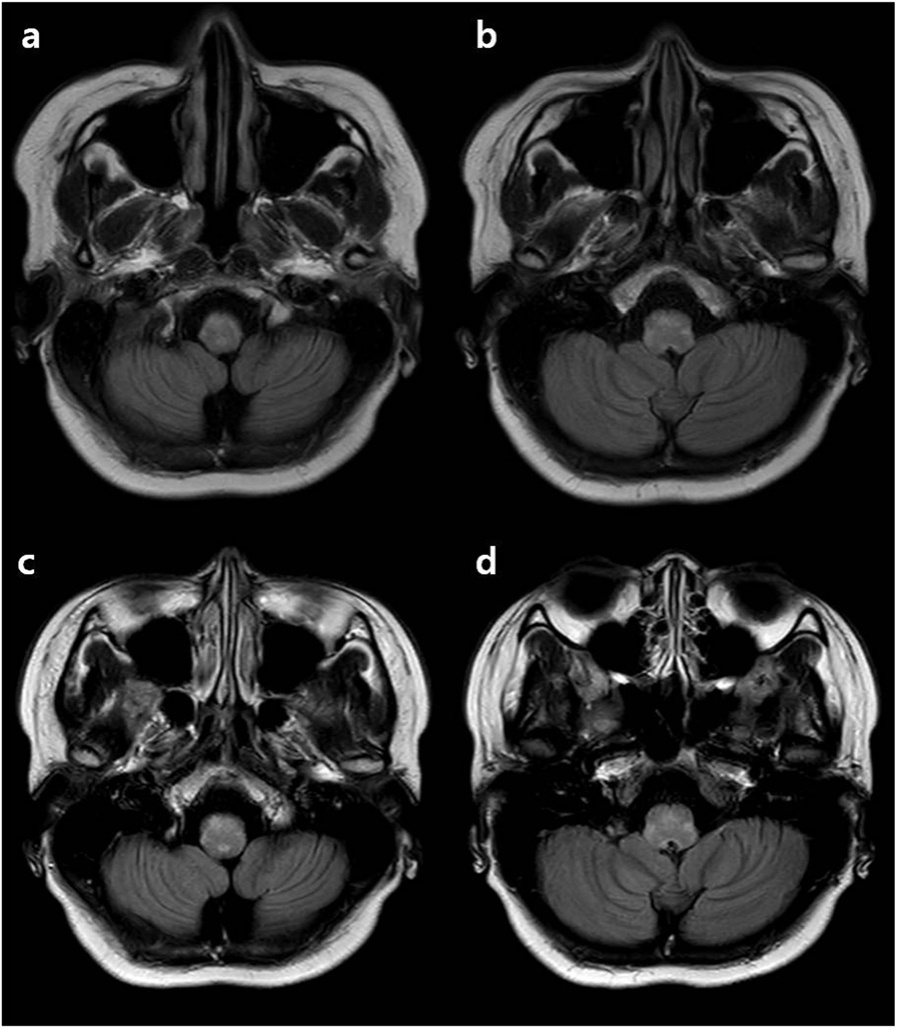
**a**, **b**, **c**, **d** Brain MRI taken at 1 week after admission (**a**, **b**) Fluid attenuated inversion recovery (FLAIR) MRI showed high signal lesions in the medulla oblongata. (**c**, **d**) FLAIR with contrast enhancement MRI showed mild enhancement in the same area

With suspicion of nausea, vomiting, and myoclonus caused by medullary lesion, a brain MRI was done at 1 week after stopping ECMO. The brain MRI showed high signal lesions in the medulla oblongata on the T2 and fluid attenuated inversion recovery (FLAIR) image (Fig. 2a,b,c,d). The cerebrospinal fluid (CSF) analysis was normal. Fluorescent antinuclear antibody (FANA) was positive with a homogenous pattern, but anti-dsDNA antibodies and Anti Ro/La-Ab were negative. There was no evidence of lupus or paraneoplastic syndrome in the clinical examination and abdomen and chest CT scan. Acute disseminated encephalomyelitis was the initial diagnosis for the medullary lesion and myoclonus. Her initial symptoms including nausea, vomiting, myoclonus, and cough were rapidly improved after the introduction of ECMO and hydrocortisone. And remaining myoclonus was completely resolved by clonazepam 0.5 mg a day for 7 days.

A predisposing neurologic event, rapid recovery of LV dysfunction and regional wall motion abnormality, ST elevation, absence of angiographic evidence of coronary disease, and pheochromocytoma were compatible with stress cardiomyopathy according to the Mayo Clinic diagnostic criteria [5].

**Fig. 3:**
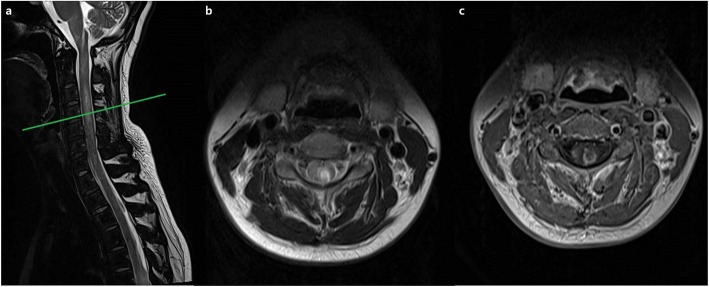
**a**, **b**, **c** Spine MRI taken at the second neurologic attack. (**a**) Sagittal spine MRI showing longitudinal extensive transverse myelitis from the medulla oblongata to the thoracic spine level on the T2-weighted image. Axial T2-weighted MRI (**b**) and T1-weighted MRI with contrast enhancement (**c**) at C4 (mark by the green line)

At around 1 month after discharge, sudden hypoesthesia developed on her left hemibody. Spine and brain MRI revealed longitudinal extensive transverse myelitis (LETM), ranging from the medulla to the thoracic spinal cord (Fig. 3a,b,c). Aquaporin-4 antibody (AQP4-Ab) was positive in a cell-based indirect immunofluorescence assay [6]. The immunoglobulin G index was elevated, and oligoclonal bands were negative. Other autoimmune diseases and infectious diseases causing LETM did not match the diagnostic criteria. She was diagnosed with NMO spectrum disorder with AQP4-IgG. Treatment with intravenous methylprednisolone 1 g a day was maintained for 7 consecutive days. After this, her neurologic symptoms were much improved. Although azathioprine 100 mg was prescribed to prevent the recurrence of NMOSD, LETM was recurred at around 2 months after the first relapse. There was no more recurrence until now for 10 months since rituximab administration.

## Discussion and conclusion

Stress cardiomyopathy is an acute cardiac condition caused by intense emotional or physical stress leading to severe but reversible cardiac dysfunction [7]. The case presented here is of a young woman without any underlying disease who, following a stressful event – medullary lesion by NMO – developed a clinical, electrocardiographic, and laboratory abnormality like ischemic heart disease. However, the absence of angiographic evidence for the coronary angiography and pheochromocytoma, the rapid reversibility of the LV regional wall motion without apical involvement, the elevation in cardiac troponin levels, and the myocardial edema suggested a diagnosis of stress cardiomyopathy, even though a biopsy of the myocardium was not done.

Although a catastrophic cardiopulmonary event like stress cardiomyopathy is very rare in CNS demyelinating diseases, several case series associated with MS have been reported [1–4]. One case series by Androdias et al. reported that all patients presented a demyelinating lesion in the medulla oblongata [4]. Only one case report with an NMO patient presenting with stress cardiomyopathy described medullary lesion involvement [8]. Likewise, the case with NMO presented here showed a demyelinating lesion in the medulla oblongata with fulminant cardiopulmonary distress, even ECMO therapy was required.

Although the pathogenesis of stress cardiomyopathy remains unclear, the predisposition of neurologic or psychiatric disorders was found very frequently in around 85% of stress cardiomyopathy [9]. The complex interaction between the heart and the brain – the heart-brain axis – may be explained as follows: the autonomic nervous system in the CNS can control cardiovascular function [10]. The dysregulation of autonomic nervous systems such as the solitary nucleus of the medulla oblongata, which is the primary regulatory region of the parasympathetic nervous system, may induce a catecholamine surge and in turn, may lead to left ventricular dysfunction through multiple mechanisms. This dysregulation may explain why stress cardiomyopathy occurred in MS or NMO patients with medullary lesions [11, 12].

APS is a condition with intractable nausea, vomiting, or hiccups associated with area postrema in the dorsal medulla. In this case, APS, as her initial presenting symptom, and the subsequent myoclonus of the limbs suggested a medullary lesion. APS is one of the core clinical characteristics for NMO and reported in up to 30% of the NMO patients during their illness. Most APS attacks precede inflammatory involvement of the optic nerves or spinal cord, making APS an important warning sign [13]. Unexplained nausea and vomiting preceding cardiopulmonary dysfunction could be a warning sign of APS.

Catastrophic stress cardiomyopathy is very rare in NMO, but NMO should be considered as one of the differential diagnoses in patients with APS and a cardiopulmonary event.

## Data Availability

All data generated in this study are included in their entirety in this published article. The first author can provide the original data if needed.

## Abbreviations

CNS: Central nervous system
NMO: Neuromyelitis optica
MS: Multiple sclerosis
APS: Area postrema syndrome
LVEF: Left ventricular ejection fraction
ECMO: Extracorporeal membrane oxygenation
MRI: Magnetic resonance imaging
CT: Computed tomography
CSF: Cerebrospinal fluid
FANA: Fluorescent antinuclear antibody
LETM: Longitudinal extensive transverse myelitis
AQP4-Ab: Aquaporin-4 antibody
